# Stencil Printing—A Novel Manufacturing Platform for Orodispersible Discs

**DOI:** 10.3390/pharmaceutics12010033

**Published:** 2020-01-01

**Authors:** Henrika Wickström, Rajesh Koppolu, Ermei Mäkilä, Martti Toivakka, Niklas Sandler

**Affiliations:** 1 Pharmaceutical Sciences Laboratory, Åbo Akademi University, Tykistökatu 6A, 20520 Turku, Finland; niklas.o.sandler@gmail.com; 2 Laboratory of Natural Materials Technology, Åbo Akademi University, Porthaninkatu 3, 20500 Turku, Finland; rajesh.koppolu@abo.fi (R.K.); martti.toivakka@abo.fi (M.T.); 3 Laboratory of Industrial Physics, University of Turku, Vesilinnantie 5, 20500 Turku, Finland; emmaki@utu.fi

**Keywords:** stencil printing, pharmacoprinting, orodispersible discs, orodisperible films

## Abstract

Stencil printing is a commonly used printing method, but it has not previously been used for production of pharmaceuticals. The aim of this study was to explore whether stencil printing of drug containing polymer inks could be used to manufacture flexible dosage forms with acceptable mass and content uniformity. Formulation development was supported by physicochemical characterization of the inks and final dosage forms. The printing of haloperidol (HAL) discs was performed using a prototype stencil printer. Ink development comprised of investigations of ink rheology in combination with printability assessment. The results show that stencil printing can be used to manufacture HAL doses in the therapeutic treatment range for 6–17 year-old children. The therapeutic HAL dose was achieved for the discs consisting of 16% of hydroxypropyl methylcellulose (HPMC) and 1% of lactic acid (LA). The formulation pH remained above pH 4 and the results imply that the drug was amorphous. Linear dose escalation was achieved by an increase in aperture area of the print pattern, while keeping the stencil thickness fixed. Disintegration times of the orodispersible discs printed with 250 and 500 µm thick stencils were below 30 s. In conclusion, stencil printing shows potential as a manufacturing method of pharmaceuticals.

## 1. Introduction

Pharmaceuticals are predominantly produced according to a centralized and time-consuming batch processing approach [1]. This supply chain model allows production of only a few dose strengths in large volumes, which for the blockbuster drugs are chosen based on population level information [2]. Challenges may arise if a patient is treated with an active pharmaceutical ingredient (API) with a narrow therapeutic window or a varying pharmacokinetic or pharmacodynamic profile. In these cases, patients would benefit from a more personalized dosing tailored according to the patient’s age, weight, body surface, gender, genetic profile, or treatment response [3,4]. A more pull-driven and personalized production of medicines could be possible if printing technologies would be utilized. Decentralized manufacturing of doses could result in added value when treating patients with medicines where tailoring and monitoring of the dose is critical. Printing technologies have for instance shown to allow simultaneous personalized dose preparation and identification [5].

Orodispersible films (ODFs) that disintegrate in the mouth have been developed to ease the administration of medicines to children and elderly that have difficulty swallowing pills [6]. Orodispersible films are conventionally manufactured using solvent casting and the formulation development is usually done on a smaller scale [7]. However, the film composition and the processing conditions need to be tuned when the production is moved from a laboratory scale to a continuous manufacturing process [8]. When manufacturing ODFs by solvent casting, the dosing can be adjusted by varying the wet film thickness during the manufacturing process or by varying the API content of the solution [9]. Dosing is achieved by cutting different areas of the film. The cutting is a critical step and it might also lead to product waste. A dosing device, which would improve the dosing flexibility, was developed to address this issue [10]. Electrospinning is another method, which has been utilized to produce ODFs [11]. Dosing flexibility is similarly achieved by cutting different areas of the electrospun film.

Impact, non-impact, and 3D printing technologies have been investigated as potential manufacturing methods of ODFs. If doses are manufactured utilizing printing technologies the critical dose cutting step is eliminated. Single dose units have either been made by depositing a drug containing ink formulation onto a placebo ODF or drug formulations directly onto a release liner or packaging material foil. High viscous solutions have been deposited onto ODFs by flexography (impact method) [12]. Low viscous drug solutions and suspensions have been deposited onto placebo ODFs by thermal, piezoelectric, and solenoid valve-based inkjet technology (non-impact methods) [13,14,15,16,17]. Especially inkjet printing has shown to allow accurate deposition of one or more APIs according to a digital design. Single unit ODF doses have been prepared by extruding a semi-solid ink formulation through a syringe using pressure assisted 3D printing according to a digital design [18]. Hot-melt extrusion has also been used to formulate solid filaments used in fused deposition modeling (FDM) 3D printers to produce ODFs [19]. Hot-melt ram extrusion 3D printing, which combines extrusion and printing, has also been explored to produce ODFs [20]. 

In solvent casting, flexographic, inkjet, and extrusion printing different solvents are used in the ink formulation, and consequently the formulations need to be dried. The use of organic solvents and the drying step has shown to have an impact on the mechanical properties of the ODFs [8]. Furthermore, the manufacturing method has also shown to have an impact on the mechanical properties; the ODFs made by solvent casting were more durable compared to ODFs prepared by FDM [19]. When considering using hot-melt extrusion as a manufacturing method of ODFs one needs to ensure that the API does not degrade by the heat of the manufacturing step. The manufacturing method needs to be chosen considering the physicochemical properties of the drug and the dosing range/flexibility needed.

**Figure 1:**
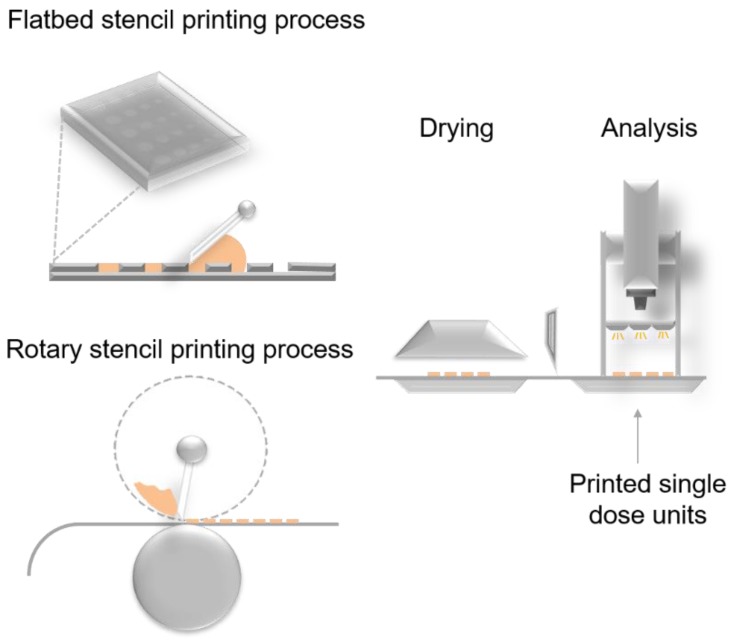
Planar and rotary stencil printing processes.

Stencil printing is a potential manufacturing method of ODFs or orodispersible discs that has not been explored before. Previously in other industries the method has shown to be suited as both a point-of-need and low-cost high-throughput manufacturing process [21,22]. Stencil printing is an impact printing method that enables pattern transfer mediated by a stencil (Figure 1). Stencil printing is a variant of screen printing in which the ink is distributed through the open pores of a patterned mesh/screen. In stencil printing the ink is passed through the stencil apertures with the help of a blade or a squeegee and can either be built as a flatbed or rotary printing process. Stencil printing has lately been utilized to manufacture wearable electronics with resolutions reaching from mm to a few µm [23,24,25]. The inks that have been used for stencil printing of electronics are solder pastes, which contain metal particles of specific particle size ranges, and the rheological properties of the pastes have been investigated with regards to stencil printability [26,27]. In general, screen and stencil printing technologies have been utilized in various fields, since it allows printing onto various materials (i.e., textiles, metal, plastics, ceramics, and paper) [28].

The suitability of producing pharmaceuticals using stencil printing has not been studied before. Thus, this article will give insight about the potential of stencil printing of drug containing polymer inks in the manufacture of personalized dosage forms. Furthermore, ink formulation properties and factors affecting the printing process are investigated and discussed. 

## 2. Materials and Methods

### 2.1. Stencil Printing Set-Up

**Figure 2:**
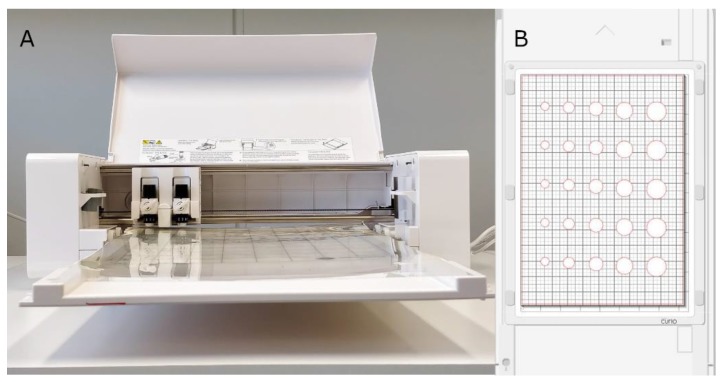
(**A**) A Silhouette Curio crafting cutter machine and (**B**) stencil aperture design.

The stencil printing can be built as a batchwise (flatbed) or a continuous (rotary) printing process (Figure 1) [28]. Critical variables affecting the stencil printing process have previously been identified and divided into the following categories: stencil, substrate, ink, printer, and environment [26]. In this proof of concept study prototype flatbed printer was used. The batchwise stencil printing set-up consisted of a drawdown coater (K202, RK Print-coat instruments Ltd., Royston, UK), a blade holder connected to a rod, a blade, and a frame. A Silhouette Curio crafting cutter (Silhouette America Inc., Lehi, UT, USA) was used to make stencils out of polyester films (125 µm; Melinex, Dupont Teijin Films, Cheste, VA, USA) and Teflon (PTFE Etraflon film) films (Figure 2A). Disc, square, and teardrop geometries were designed using the Silhouette studio v3. Software (Silhouette America Inc.) and the shapes were cut out from the films. The cut polyester stencil films were glued together to make 250, 500, 750, and 1000 µm thick stencils. The Teflon films were acquired with thicknesses of 250, 500, 1000, and 1500 µm. Stencils with varying stencil area (Ø 10.8, 14.4, 18.0, 21.6, and 25.2 mm) were only prepared for the disc geometry (Figure 2B). 

### 2.2. Ink Formulation

Hydroxypropyl methylcellulose (HPMC) inks with polymer contents of 12–18% were prepared as placebo formulations. HPMC (Methocel E5 Premium LV, Dow, Bomlitz, Germany) was dissolved in a deionized water and ethanol (Etax Aa 99.5%, Altia Oyj, Rajamäki, Finland) solvent mixture (1:1, *v/v*) using high speed mixing. Glycerol (CAS 200-289-5, Fagron GmbH & Co. KG, Glinde, Germany) (1%) was used as a plasticizer. Erythrosin B (spirit soluble 95%, CAS 16423-68-0, Aldrich Chem. Co., Milwaukee, WI, USA) was added (1%, *v/w*) as colorant to the formulations. Formulation development was continued with the formulation that contained 16% of HPMC. Lactic acid (LA, 1%, *v/w*) (CAS 50-21-5, Sigma-Aldrich, Japan), was added to the 1:1 ethanol water solvent mixture to lower the pH and consequently allow haloperidol (HAL, CAS 52-86-8, Sigma, China) to dissolve. Table 1 lists the various combinations of ink formulations that were studied. 

### 2.3. Stencil Printing

Printing of the orodispersible discs was performed at a speed of 259 mm/min using transparency films (Folex Imaging, Clear transparent X-10.0 film, 0.100 mm) as substrate/release liner. The ink was poured onto the 250, 500, 750, and 1000 µm thick stencils and was distributed with the help of a blade. Each printing pass generated 15 doses. The printed discs were dried overnight in an oven (T = 25 °C, RH % = 60).

### 2.4. Rheology

The dynamic viscosity and the thixotropic flow (time dependent recovery) of the inks were measured using a rheometer (Physica MCR 300, Paar Physica, Graz, Austria) connected with a thermostated bath and a temperature control unit (Techne RB-12A & TU-16D). The cone and plate measurement geometry (50 mm diameter and 2° angle) was used for the measurements. Prior to the measurements the samples were preconditioned for 1 min at a shear rate of 100 s^−1^ and then let to rest for 2 min. A shear rate ramp ranging from 0.1 to 1000 s^−1^ was applied to the samples (*n* = 3) at 25 °C. Thixotropy was evaluated with a step test in which the time dependent viscosity recovery is observed after applying a high-shear rate (500 s^−1^) step change to a low constant shear rate 0.1 s^−1^. Formulations containing 16% of HPMC were further studied.

### 2.5. Visual Print Evaluation

The spreading of inks HPMC 12–18% was studied by capturing images of discs dried overnight at 25 °C and relative humidity of 60%. The discs (Ø 18 mm) were printed with 250, 500, and 1000 µm thick stencils onto transparency film substrates. Images were captured using a mobile phone camera (OnePlus5T OnePlus Technology Ltd., Shenzhen, China) at a fixed height. Spreading of the inks with different polymer content on the substrates was analyzed based on the captured images using the ImageJ software. Before the image analysis was performed, the hue and saturation values of the image were adjusted, the scale was set, and the printed area with 12 discs was selected.

### 2.6. pH

The pH of the ink formulations and the printed discs was measured using a pH meter (FE20, Mettler Toledo AG, Schwerzenbach, Switzerland). For the surface pH of the discs (500 µm stencil, Ø 18 mm) 1 mL of distilled water was used to wet the surface and provide adequate contact with the electrode. The reading was taken after allowing the pH electrode to equilibrate for 1 min on the surface of the discs.

### 2.7. Disintegration

The test system consisted of a sample holder clamp to which the printed disc was attached. A smaller clip of 3 g was fixed to the bottom of the disc as a weight. The disintegration tests were performed using a static test set-up by immersing half of the disc (*n* = 10) and the weight into a beaker of 500 mL distilled water (37.0 ± 0.5 °C). The endpoint was recorded when the 3-gram clip reached the bottom of the disintegration beaker. The tests were performed under ambient conditions of samples conditioned overnight in 25 °C and 60% relative humidity. Discs (Ø 18 mm) printed using HPMC 12–18% placebo inks and 250, 500, 750, and 1000 µm thick stencils were analyzed.

### 2.8. Drug Assay

A HPLC (LaChrome, Merck Hitachi, Tokyo, Japan) system (Interface D7100, Pump L-7100, Autosampler L-7200, Detector L-7450, Shimadzu column oven CTO-10) was used for the quantification of the printed HAL doses. The separation was performed using an Intersil ODS-3.5 µm, 4.6 × 150 mm column (GL Sciences, Tokyo, Japan) with a pre-column (Guard Column E 10 mm) attached. The mobile phase consisted of 0.05% TFA in deionized water (CAS 76-05-1, Trifluoroacetic acid, Sigma-Aldrich, Germany) and 0.05% TFA in ACN (CAS 75-05-8, Acetonitrile, HPLC grade, Fisher Chemicals, Loughborough, UK) (65:35, *v/v*). The flow rate was 1 mL/min, injection volume was 10 µL, and detection wavelength was set at 243 nm. 

### 2.9. Uniformity of Mass of Single-Dose Preparations

The stencil printed discs of different sizes (Ø 10.8, 14.4, 18.0, 21.6, and 25.2 mm) were conditioned for 12 h at 25 °C and 60% relative humidity before being weighed. A total of 20 units were chosen at random and weighed individually.

### 2.10. Uniformity of Content of Single Dose Preparations

The content uniformity of the stencil printed discs of different sizes (Ø 10.8, 14.4, 18.0, 21.6, and 25.2 mm) was determined (*n* = 10) in 100 mL of 1% lactic acid deionized water/EtOH solution (1:1, *v/v*) for 2 h at 150 rpm. Samples were withdrawn and analyzed as such according to the drug assay described above.

### 2.11. Polarized Light Microscopy

The stencil printed discs were imaged using a microscope with transmitted polarized light (Leica DM IRB microskopie and Systeme GmbH, Wezlar, Germany) with ×10/0.3 magnification (PL fluotar 506000). Images of starting materials and printed discs were captured using a OnePlus 5T mobile phone camera.

### 2.12. X-ray Powder Diffraction

The crystallinity of the printed disc components was studied with X-ray diffraction (XRD). The samples were placed on zero-background holders and scanned with PANalytical Empyrean diffractometer (Malvern Panalytical B.V., Almelo, the Netherlands) in θ/θ Bragg-Brentano geometry, using Cu Kα radiation with a PIXcel3D detector in scanning line mode. The scan was done for 2θ in a range of 5–50° with a step size of 0.013° using a step time of 60 s. The incident beam optics consisted of a 0.04 rad Soller slit and a fixed 1/4° divergence slit, while the diffracted beam optics included a 0.04 rad Soller slit.

### 2.13. Differential Scanning Calorimetry (DSC)

DSC measurements were carried out using a conventional DSC (Q 2000, TA instruments, USA). The printed samples were measured in Tzero aluminum pans with perforated (discs) and intact (raw materials) lids. Thermograms of the discs were recorded in a heat/isothermal/cool/heat cycle. The samples (5.1 ± 0.2 mg) were heated up to 100 °C with a rate of 10 °C/min, held at 100 °C at 5 min, cooled down to −40 °C at a rate of 10 °C/min and heated up to 200 °C using a rate of 10 °C/min. Thermograms of raw materials (5.03 ± 0.2 mg) were recorded as a ramp from 0 to 200 °C using a rate of 10°/min. The DSC was calibrated using sapphire crystals and indium. 

### 2.14. Fourier Transform Infrared Spectroscopy (FTIR)

The FTIR spectra of the samples were obtained using Bruker Invenio R spectrometer (Bruker Optics GmbH, Ettlingen, Germany), equipped with a PA301 photoacoustic detector (Gasera Oy, Turku, Finland) using dried air as the carrier gas. 

## 3. Results

### 3.1. Stencil Preparation

The polyester film (250 µm) was the material with the most favorable characteristics to be used as stencil material. The apertures and geometries were easily designed and cut from the polyester films. Since the stencil thickness and the aperture area of the stencils were the dose defining units, the possibility to vary these factors was of importance. Thicker stencils were produced by attaching 250 µm films on top of each other to make 250, 500, 750, and 1000 µm thick stencils. The stencils made of Teflon were not suitable because the thinnest films (250 and 500 µm) were not rigid enough, which caused challenges during the aperture emptying when the stencil was removed and the thickest (1000 and 1500 µm) were not flat enough. Thus, polyester was the best option as stencil material. 

### 3.2. Ink Formulation Development

The suitability of producing variable dose strengths using a flatbed stencil printing process was studied. HAL was chosen as a model drug. The drug is used for treatment of schizophrenia and the therapeutic dose for 6–17 year-old children is 0.5–3 mg, while it is up to 10 mg once a day for adults [29]. HAL is known to be a CYP2D6 substrate and polymorphism of the liver enzyme between individuals has a large impact on the pharmacokinetics of the drug [30]. Genotype identification of the CYP2D6 substrate is estimated to be beneficial for 30–40% of patients, when selecting the active pharmaceutical ingredient or customizing the dose for an individual. Consequently, patients could benefit from having personalized doses printed of this specific drug.

HPMC was chosen as a matrix former since it is soluble in water and ethanol mixtures and remains stable in pH between 3–11. HAL is practically insoluble in water, slightly soluble in ethanol, and freely soluble in dilute acids [31]. Lactic acid (1%) was added to the formulation to enable the drug to dissolve. HAL is known to be stable in aqueous lactic acid solutions at room temperature [32] and known to be a saliva stimulant, which is of relevance when formulating an orodispersible drug formulation [33]. Erythrosine was chosen as colorant due to its good solubility in ethanol and glycerol was added as a plasticizer. The ink formulation is shown in Table 2.

### 3.3. Ink Rheology and Printability

**Figure 3:**
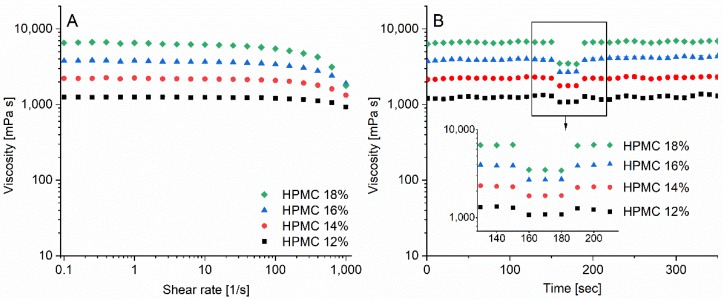
(**A**) Shear ramp of formulations containing 12–18% polymer (*n* = 3 ± std), (**B**) thixotropic flow measurements (shear rate: 0.1, shear rate: 500, shear rate: 0.1 s^−1^, *n* = 3 ± std) for hydroxypropyl methylcellulose (HPMC) 12–18%.

**Figure 4:**
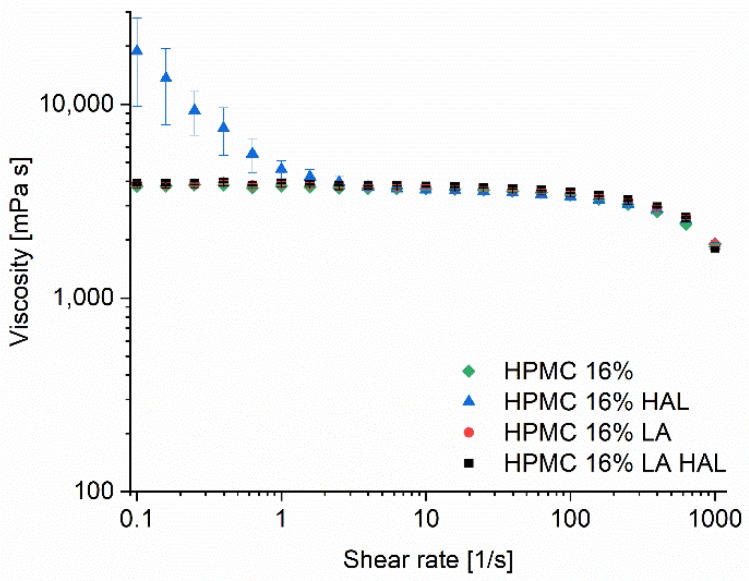
Shear ramp of HPMC 16% formulations (HAL = haloperidol, LA = lactic acid).

The rheological characteristics of the inks were determined by applying a shear ramp on the samples and by monitoring the time dependent recovery of the samples after application of a step change in shear rate. The HPMC 12–18% inks showed all Newtonian behavior between shear rates 0.1 and 100 s^−1^. However, at higher shear rates the inks showed shear-thinning behavior. All inks had higher low shear viscosities than 1000 mPas; at a shear rate of 10 s^−1^ the viscosity of the inks HPMC 12, 14, 16, and 18% were 1240, 2177, 3660, and 6190 mPas, respectively (Figure 3A). The thixotropic flow of the inks was also investigated. All inks recovered quickly to their original viscosity levels after an applied high shear rate step of 500 s^−1^ for 30 s (Figure 3B). In general, the viscosity of the ink should be low enough to allow the blade to press and fill the ink through the apertures of the stencil, but high enough to retain its geometry when the stencil is removed. Ink formulations with HAL dissolved (HPMC 16% LA HAL) and dispersed (HPMC 16% HAL) were prepared, which enabled comparison of the rheological properties of inks with and without particles. The dispersed particles had an impact on the viscosity at low shear rates, while the HPMC 16% LA HAL formulation showed similar behavior as the HPMC 16% and HPMC 16% LA formulations with the same polymer content (Figure 4). 

**Figure 5:**
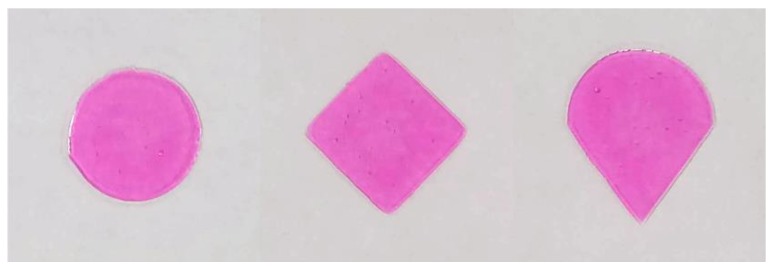
Stencil printed discs of 3 geometries using ink formulation HPMC 16% (500 µm stencil height).

The printability was evaluated in terms of aperture filling of different geometries and spreading of the ink onto the release liner. The aperture geometry was evaluated at a fixed printing speed (259 mm/min) and using a 500 µm stencil with disc, square, and teardrop apertures. No clear difference in aperture filling was observed between geometries when ink formulation HPMC 16% was printed under the above-mentioned conditions (Figure 5). However, at higher speeds differences could possibly be distinguished. It has been reported that the aperture geometry should be rotated 45° to the printing direction to ensure uniform filling of the apertures [34]. Taking this into account, the square and teardrop (printed from the sharper direction) should be preferred. However, the circular geometry is less prone to slumping, meaning that it has a greater ability to retain the printed shape.

**Figure 6:**
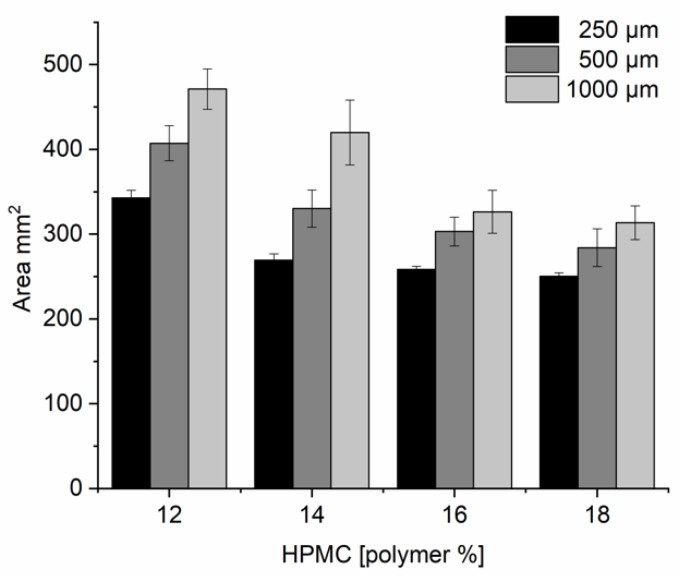
Area from printed Ø 18 mm discs analyzed using ImageJ (*n* = 12) for formulations with increasing polymer content and stencil height.

Next, the spreading of the inks onto the release liner was studied. Inks were printed using stencils with circular apertures (Ø 18 mm) and stencil thicknesses of 250, 500, and 1000 µm. The spreading of the ink onto the release liner was evaluated based on image analysis done after allowing the samples to dry for 24 h at 25 °C and 60% relative humidity. The lower the ink viscosity and the higher the stencil thickness, the more the ink spread on the transparency film/release liner (Figure 6). Formulations containing less than 16% of HPMC were seen to spread more than 15% from the original stencil aperture area of 254.57 mm^2^. Due to the low viscosity of the inks some spreading was expected. Higher print quality could also be achieved by making fine pitch stencils by laser cutting.

### 3.4. Disintegration of Discs

**Figure 7:**
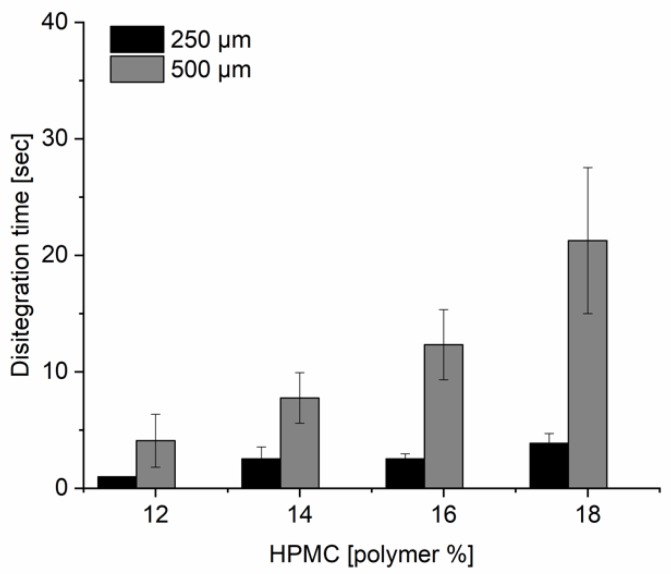
Disintegration time (s) for 18 mm disc formulations (*n* = 10) with increasing polymer content (12–18%) and 250 and 500 µm thick stencils.

The disintegration times of the discs (Ø 18 mm) containing 12–18% of HPMC and printed using 250 µm stencil were below 4 s and for 500 µm stencils below 28 s (Figure 7). The disintegration results of the discs prepared using the 500 µm stencil fulfilled the specification threshold of 30 s for orodispersible tablets. The European Pharmacopeia 9th edition (Ph. Eur. 9th) does not contain any specifications regarding disintegration times of ODFs. The disintegration times for the 12, 14, 16, and 18% of HPMC discs printed with the thickest stencil 1000 µm were 29 ± 5, 49 ± 18, 140 ± 37, and 200 ± 30 s, respectively. Based on these disintegration results and on the results from the spreading tests in Section 3.2, the formulation containing 16% of HPMC was further studied.

### 3.5. pH of Ink Formulations and Disc Surfaces

The impact of HAL and LA on the formulation pH was investigated. LA lowered the pH of the ink formulation to 3–4, whereas the addition of HAL increased the pH. The surface pH of the stencil printed discs was determined to be above 4 (Table 3). ODFs with a surface pH of 4.5–6.5 have been investigated not to cause local irritation of the mucosa in the mouth [35]. Drug delivery of slightly acidic ODFs is less likely to cause harm the mucosa compared to mucoadhesive buccal films that are attached for a longer time to the mucosal membrane [36].

### 3.6. Quantification of Haloperidol by HPLC

HAL was detected using HPLC. The method used showed to be linear (R^2^ = 0.999) within a concentration range of 1–50 µg/mL. The detection limit (LOD) was 0.38 µg/mL and quantification limit (LOQ) was 1.15 µg/mL. The retention time of HAL was 4.5 min (capacity factor 2). The method was seen to be reproducible and the samples were seen to remain stable (Supplementary Materials Table S1).

### 3.7. Uniformity of Mass and Content

The printed discs fulfilled the requirements of mass uniformity stated in Ph. Eur. 9th. No mass unit deviated more than 10% from the average mass. The printed discs also fulfilled the content uniformity requirements. Each individual content was between 85–115% of the average content. Linear dose escalation was seen for the discs with various aperture areas printed with a 500 µm stencil (R^2^ = 0.9961) (Table 4). The mass and dose escalation were not precisely doubled as the stencil thickness increased in the following range: 250–500–1000 µm. The amount of paste transferred is dependent on the aperture size and pressure [37]. Repetition of the dose escalation study could be done in the future with a stencil printer where the pressure could be set and monitored. Furthermore, the removal of the stencil should also be automated to minimize human error. 

### 3.8. Polarized Light Microscopy

**Figure 8:**
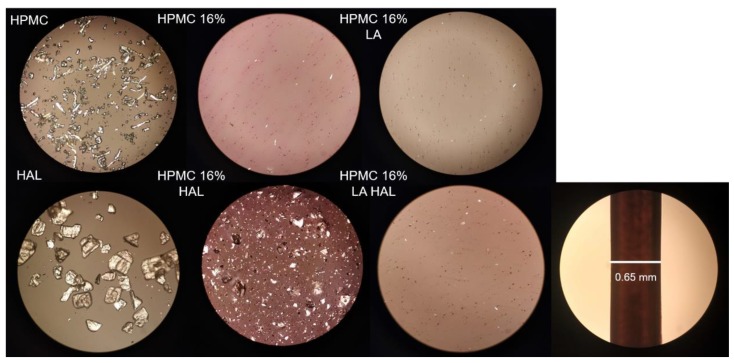
Polarized light microscopy of raw materials HPMC and haloperidol (HAL) and printed discs HPMC 16%, HPMC 16% lactic acid (LA), HPMC 16% LA HAL, and HPMC 16% HAL (magnification ×10), stencil height 500 µm.

The starting material as well as printed discs were studied under the polarized light microscope (Figure 8). The crystal morphology of HPMC was acicular, while the HAL was platy. The particle size of HAL was larger compared to the HPMC polymer. HPMC was almost entirely dissolved in the solvent mixtures with and without LA (HPMC 16% and HPMC 16% LA). The pH drop caused by LA enabled HAL to dissolve, while it remained dispersed in the neutral polymer matrix (HPMC 16% HAL). Lower pH was shown to lighten the red color of erythrosine, which is due to its predominantly unionized form at a pH below 4.5 [38]. Consequently, the colorant was less soluble and provided lower color intensity in the solvent mixtures containing LA.

### 3.9. X-ray Diffraction

**Figure 9:**
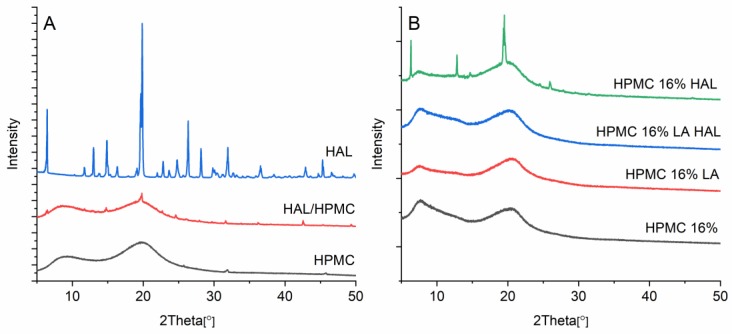
(**A**) X-ray diffractogram of powders HAL, HPMC, and physical mixture HAL/HPMC (1/16) and (**B**) printed discs of HPMC 16%, HPMC 16% LA, HPMC 16% LA HAL, and HPMC 16% HAL.

The printed formulation with HAL dispersed in the polymer matrix (HPMC 16% HAL) showed small diffraction peaks at 12.8, 14.9, 19.9, and 25.9 (Figure 9). These peaks correspond to those observed in the initial crystalline HAL. The HPMC powder was amorphous as suggested by the broad halos near 10° and 20° with a small diffraction peak at 31.9°, which is typical for the polymer. The physical mixture of HAL/HPMC (1/16) correspondingly showed the attenuated reflections related to the pure API on the amorphous HPMC background, suggesting limited interaction between the API and the polymer when only mixed. The printed formulation HPMC 16% LA HAL was amorphous. Consequently, the small addition of LA made the solvent mixture more acidic, which in turn enabled the drug to dissolve.

### 3.10. Differential Scanning Calorimetry (DSC)

**Figure 10:**
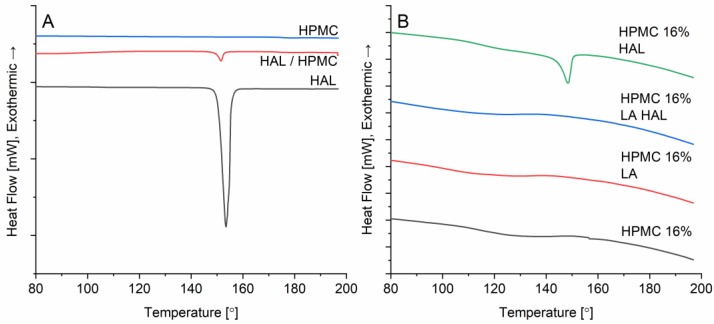
Thermograms of the (**A**) pure components and their physical mixture in 1:16 w/w ratio (HAL/HPMC) and (**B**) printed discs of HPMC 16%, HPMC 16% LA, HPMC 16% LA HAL, and HPMC 16% HAL, (stencil thickness 500 µm).

For reference purposes, the thermal behavior of the pure HAL and HPMC were analyzed with DSC. The crystalline HAL expectedly underwent melting at 150.8 °C with an enthalpy of fusion, ΔHfus = 140.5 J/g (Figure 10). The pure HPMC, as the XRD diffractogram suggested, did not indicate any phase transitions in the utilized temperature range, showing only a glass transition occurring at ca. 174–175 °C. Only a slight shift in the melting onset of HAL was observed for the physical mixture, as the phase transition began at 149.5 °C. The DSC results from the ink formulations supported the observations made with the XRD. The only disc where crystalline HAL could be detected was the HPMC 16% HAL with a melting point of 144.6 °C and heat of fusion of 2.78 J/g.

### 3.11. Fourier Transform Infrared Spectroscopy

**Figure 11:**
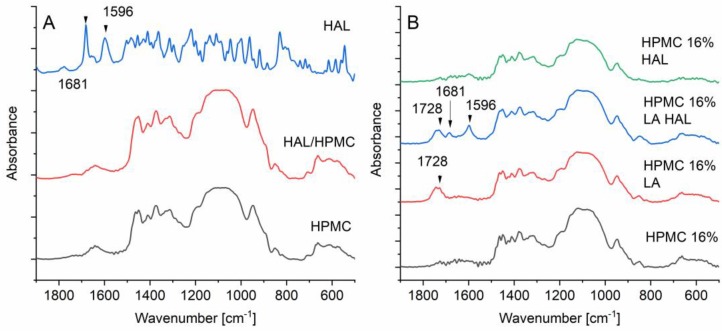
Infrared spectra of (**A**) pure HAL, HPMC, and physical mixture of HAL/HPMC (1/16) and (**B**) printed discs of HPMC 16%, HPMC 16% LA, HPMC 16% LA HAL, and HPMC 16% HAL.

The carbonyl stretch in HAL at 1681 cm^−1^ could be distinguished in the printed HPMC 16% LA HAL discs where the drug was dissolved in the ink (Figure 11) [39]. However, the peak was not detected for the HPMC 16% HAL discs with crystals. Because the XRD and DSC results showed amorphization of the HPMC 16% LA HAL, one would expect to see a shift in the carbonyl group of the HAL due to hydrogen bond formation. However, this could not be confirmed with the FTIR measurements. The peak at 1728 cm^−1^ (carbonyl stretch of the acid) belonging to lactic acid was distinguished for printed discs of the HPMC 16% LA and HPMC 16% LA HAL formulations [40].

## 4. Discussion

The suitability of producing pharmaceuticals using stencil printing was explored. Polymer-based ink formulations were developed and printed according to the predefined geometries cut from the stencil to prepare personalized doses of HAL. The stencils were the dose defining units; different doses were varied by aperture areas and stencil heights. The higher the viscosity of the ink formulation, the less spreading of the solution occurred after printing. Thus, ink solutions with dynamic viscosities >3000 mPas (shear rate 10 s^−1^) were the most favorable formulations for stencil printing. Formulations with dynamic viscosities of at least 1000 mPas (shear rate 6 s^−1^) have been successfully used to produce ODFs by solvent casting [8]. The viscosity of the ink formulation is of major importance in solvent casting, since it affects the API content per cm^2^. It is less critical in stencil printing, since the dose and the volume printed is defined by the aperture of the stencil. 

This study shows that a pull-driven and batchwise production of personalized doses utilizing stencil printing technology and a polymer-based ink formulation was possible. A printing speed of 259 mm/min was used to manufacture 15 doses at a time, while solvent casting speeds of 17–339 mm/min have been used to manufacture ODFs [8]. The solvent cast film needs to be cut to make personalized doses. Cutting, which has been defined as a critical process parameter, might also cause drug waste. Thus, printing is more suited for the production for personalized doses.

The placebo discs printed using the 250 and 500 µm thick stencils disintegrated completely within 5 and 30 s, respectively. These results are in line with disintegration results of HPMC ODFs found in literature [41]. 

The robustness of the stencil printing process could be improved by automating the stencil movement and ink distribution steps. Addition of a drying unit to the plate on which the printing is performed and optimization of the drying time would speed up the process. Since the ink formulations used in this study were comparable with solvent casting formulations, drying was also considered as the time limiting step for the stencil printing process. Automated batchwise printing, drying, and analysis unit could be used for manufacturing personalized doses. 

Alternatively, the production capacity of stencil printing could be improved if the solvent amount of the ink formulation would be reduced. Particles are often included in the inks or pastes used for manufacturing of electronics using stencil printing technology and the pastes show usually non-Newtonian behavior and have higher viscosities [26]. Adjustment of the solder paste rheology have been reported to enable high print resolution [23,24,25]. The rheology can be adjusted by varying the particle size, distribution, and shape, as well as the solvents and the viscosity controlling agents [42]. Development of a paste with higher solid content of an API with small particle size and narrow size distribution could be done in the future. The advantages of the orodispersible dosage form could remain by printing a pastier ink onto a placebo ODF or onto a porous substrate to ease administration. However, ink optimization would need to be conducted to ensure accurate print quality and ink compatibility with the substrate. This alternative could suit continuous and high-throughput manufacturing of stencil printed dosage forms with a more restricted dosing flexibility. However, to develop a robust stencil printing process that enables the production of dosage forms that fulfill the quality requirements set by regulatory authorities entails interplay among engineering, formulation development, and quality assurance.

## 5. Conclusions

This study demonstrated that a polymer-based HAL ink formulation could be printed using a batchwise stencil printing process to prepare orodispersible HAL dosage forms for children within a therapeutic dose range and fulfilling both mass and content uniformity requirements. The polymer-based inks were Newtonian at shear rates below 100 s^−1^ and recovered quickly to the original viscosity level after application of a high shear rate step. X-ray and DSC results suggest that HAL was amorphous for the printed formulation HPMC 16% LA HAL and crystalline for the printed formulation HPMC 16% HAL, without LA. 

## Figures and Tables

**Table 1:** The compositions of the formulations used.

Formulations	Lactic Acid (LA)	Haloperidol (HAL)
HPMC 16%	−	−
HPMC 16% LA	+	−
HPMC 16% LA HAL	+	+
HPMC 16% HAL	−	+

**Table 2:** Ink formulation.

Ingredient	Function	Quantity (%)
HAL	API	1
HPMC E5	Matrix former	12–18
Erythrosine	Colorant	1
Glycerol	Plasticizer	3.5
Ethanol	Solvent	50
Lactic acid 1% (aq.)	Solvent, pH modifier, saliva stimulant	Ad 100

**Table 3:** Ink formulation and disc surface pH (average ± standard deviation) (21.5 ± 1 °C).

Formulation	Ink Formulation (*n* = 3)	Surface pH (*n* = 3)
HPMC 16%	7.10 ± 0.02	6.02 ± 0.28
HPMC 16% LA	3.23 ± 0.06	4.18 ± 0.45
HPMC 16% LA HAL	4.24 ± 0.09	4.08 ± 0.15
HPMC 16% HAL	8.00 ± 0.06	5.59 ± 0.14

**Table 4:** Uniformity of mass (*n* = 20, average ± standard deviation) and uniformity of content (*n* = 10, average ± standard deviation) of discs (Ø 10.8–25.2 mm) printed with HPMC 16% LA HAL and 500 µm stencil.

Discs (Ø mm)	Uniformity of Mass (mg ± sd)	Uniformity of Content (mg ± sd)
10.8	10.78 ± 0.20	0.49 ± 0.01
14.4	18.70 ± 0.53	0.87 ± 0.02
18.0	29.43 ± 0.83	1.43 ± 0.03
21.6	42.59 ± 1.11	1.97 ± 0.04
25.2	55.30 ± 1.34	2.56 ± 0.07

## Supplementary Materials

The following are available online at https://www.mdpi.com/1999-4923/12/1/33/s1. Table S1. Repeatability, reproducibility and sample stability of the HPLC method.
