# Supplementary material for: Stencil Printing—A Novel Manufacturing Platform for Orodispersible Discs
Source: Pharmaceutics. 2020 Jan 1;12(1):33. doi: 10.3390/pharmaceutics12010033 (PMC7023198; doi:10.3390/pharmaceutics12010033)
Supplement: Supplementary file 1 [file pharmaceutics-12-00033-s001.pdf]

pharmaceutics

Supplementary Materials: Stencil Printing—A Novel Manufacturing Platform for Orodispersible Discs

Henrika Wickström *, Rajesh Koppolu, Ermei Mäkilä, Martti Toivakka and Niklas Sandler

Table S1. Repeatability, reproducibility and sample stability of the HPLC method.

**Repeatability (*n* = 6 within 1 day)**			
Conc.[1]	10	30	50
μg/ml[2]	10.11	32.12	49.64
Std	0.06	0.12	0.18
RSD%	0.56	0.41	0.35
**Reproducibility (*n* = 12 within 3 days)**			
Conc. [1]	10	30	50
μg/ml[2]	10.13	29.92	49.93
Std	0.02	0.18	0.25
RSD%	0.22	0.58	0.5
**Sample stability (*n* = 6 within 2 days)**			
Conc. [1]	10	30	50
μg/ml[2]	10.07	30.01	49.66
Std	0.05	0.15	0.01
RSD%	0.47	0.49	0.03

[1] theoretical and [2]measured concentrations.
